# Flow Cytometric Detection of Bone Marrow Cell Populations Phenotypically Compatible with Neuroblastoma: An Exploratory Comparison with Conventional Methods

**DOI:** 10.3390/cancers18203355

**Published:** 2026-10-16

**Authors:** Karolina Bukowska-Strakova, Marta Surman, Izabela Siemińska, Aleksandra Dziewońska, Monika Rygielska, Anna Śliwińska, Aleksandra Łosiewicz, Krystyna Gałązka, Urszula Żebrowska, Maciej Siedlar, Aleksandra Wieczorek, Walentyna Balwierz

**Affiliations:** 1 Department of Clinical Immunology, Institute of Pediatrics, Jagiellonian University Medical College, 30-663 Krakow, Poland; k.bukowska-strakova@uj.edu.pl (K.B.-S.); i.gorska@uj.edu.pl (I.S.); maciej.siedlar@uj.edu.pl (M.S.); 2 Hematology and Oncology Diagnostic Laboratory, Department of Clinical Immunology, University Children’s Hospital of Krakow, 30-663 Krakow, Poland; marta.surman@usdk.pl; 3 Department of Infectious Diseases and Public Health, Faculty of Veterinary Medicine, University of Agriculture in Cracow, 31-120 Krakow, Poland; 4 Department of Clinical Biochemistry, University Children’s Hospital of Krakow, 30-663 Krakow, Poland; aleksandra.dziewonska@usdk.pl (A.D.); monika.rygielska@usdk.pl (M.R.); 5 Department of Nuclear Medicine, Children’s Memorial Health Institute, 04-730 Warsaw, Polanda.losiewicz@ipczd.pl (A.Ł.); 6 Department of Pathomorphology, Jagiellonian University Medical College, 30-663 Krakow, Poland; 7 Department of Pediatric Oncology and Hematology, University Children’s Hospital of Krakow, 30-663 Krakow, Polanda.wieczorek@uj.edu.pl (A.W.); 8 Department of Pediatric Oncology and Hematology, Institute of Pediatrics, Jagiellonian University Medical College, 30-663 Krakow, Poland

**Keywords:** neuroblastoma, bone marrow involvement, flow cytometry, cytomorphology, histopathology, 123I-MIBG

## Abstract

**Background:** Accurate evaluation of bone marrow involvement is important for staging and treatment monitoring in pediatric neuroblastoma. This study evaluated flow cytometry for detecting neuroblastoma cells in bone marrow aspirates. **Methods:** In this prospective exploratory correlative analysis, samples from 28 children were assessed through flow cytometry and cytomorphology; available histopathological and iodine-123 metaiodobenzylguanidine (MIBG) scintigraphy results were compared. Neuroblastoma cells were identified as nucleated CD56^+^CD45^−^CD36^−^CD71^−^ events. **Results:** Flow cytometry detected neuroblastoma cell populations across a broad quantitative range, including low-level populations. Agreement between flow cytometry and cytomorphology was 90.0% (Cohen’s κ = 0.56). In 16 paired aspirate-level records with at least one positive result, flow cytometric and cytomorphological percentages showed a positive Pearson correlation (r = 0.878, *p* < 0.0001), although this association was sensitive to one high-value observation. Agreement with histopathology was 92.1–94.8% (κ = 0.59–0.64), whereas agreement with MIBG scintigraphy was slight to fair (κ = 0.12–0.23). Conclusions: Flow cytometry provided rapid, quantitative detection of neuroblastoma cell populations in bone marrow aspirates. It should be considered complementary to cytomorphology and histopathology, as repeated examinations, the limited panel, and the lack of an independent reference standard preclude definitive estimates of diagnostic performance.

## Simple Summary

Neuroblastoma frequently disseminates to the bone marrow, and accurate assessment of bone marrow involvement is important for staging and treatment monitoring. In this prospective exploratory correlative analysis, flow cytometry was compared with cytomorphology, histopathology, and iodine-123 metaiodobenzylguanidine scintigraphy in bone marrow samples from 28 children with neuroblastoma. Flow cytometry provided rapid and quantitative detection of neuroblastoma cell populations in bone marrow aspirates, including populations present at low levels. However, discordant results occurred, particularly when flow cytometry was compared with histopathology or whole-body scintigraphy. These methods evaluate different biological and anatomical aspects of disease and should therefore be considered complementary. Larger, preferably multicenter, prospective studies using standardized marker panels and predefined analytical thresholds are required before flow cytometry can be incorporated into routine neuroblastoma bone marrow assessment.

## 1. Introduction

Neuroblastoma (NBL) is the most common extracranial solid tumor in children, accounting for approximately 6 to 11 cases per million annually [1]. It arises from immature neural-crest-derived precursors of the sympathetic nervous system and is typically diagnosed before the age of five years, with a median age at diagnosis of 17 months [1]. NBL presents with a wide range of clinical features influenced by tumor location, disease stage, and metastatic spread. Its clinical course is highly variable, ranging from tumors that regress spontaneously to aggressive disease. Alterations affecting ALK and RAS-MAPK signaling may support tumor growth and contribute to treatment resistance [2]. Other high-risk tumors show abnormalities in telomere maintenance, including TERT rearrangements or ATRX-associated alternative lengthening of telomeres, as well as characteristic chromosomal alterations [2,3]. Rare inherited variants in ALK or PHOX2B can also predispose to neuroblastoma [2].

Bone marrow involvement (BMI) is important for neuroblastoma staging and response evaluation [4,5,6]. In the INRG system, risk classification is based on disease stage together with age and tumor biological features; BMI contributes to this assessment but does not alone define high-risk disease [4]. Cytomorphology and histopathology of bilateral bone marrow samples remain the standard methods, complemented by immunocytochemistry where available [7,8,9,10,11]. 123I-MIBG scintigraphy provides whole-body assessment of skeletal and soft tissue disease [12,13]. Recent systematic evidence supports combining cytomorphology, trephine biopsy, immunocytochemistry, molecular assays, flow cytometry, and functional imaging, particularly because bone marrow involvement may be focal or present at a low level [8,14,15].

Flow cytometry allows for detailed analysis of cell populations based on antigen expression and may provide a rapid, objective, and quantitative assessment of bone marrow involvement [16,17,18,19,20]. By targeting markers associated with neuroblastoma cells while excluding hematopoietic and erythroid populations, it may complement conventional diagnostic methods [21]. This study aimed to evaluate the clinical utility of flow cytometry by comparing its results with cytomorphology, histopathology, and 123I-MIBG scintigraphy. The novelty of the present study lies in the fraction-specific FCM assessment of whole bone marrow and density gradient fractions, compared with cytomorphology, routine histopathology, and whole-body 123I-MIBG scintigraphy in serial bilateral bone marrow examinations.

## 2. Materials and Methods

### 2.1. Patients

The study group consisted of 28 children (7 females and 21 males; median age, 55 months; interquartile range, 22–63 months) with neuroblastoma who were hospitalized and treated at the University Children’s Hospital in Krakow. This was a prospective exploratory correlative analysis conducted within the ChIm-NB-PL phase IIa study of dinutuximab beta combined with chemotherapy in children with primary refractory, progressive, or relapsed neuroblastoma (EudraCT 2021-003832-96; NCT05272371). All consecutively enrolled patients who underwent the protocol-specified flow cytometric assessment of bone marrow aspirates between 24 March 2022 and 25 July 2025 were included in the present analysis. Flow cytometry was prospectively planned in the study protocol and was not performed as part of routine hospital diagnostics. No separate a priori sample size calculation was performed for this analysis. Although the comparison of diagnostic methods and their predictive relevance constituted secondary endpoints of the clinical study, the present analysis was not powered to provide definitive estimates of diagnostic accuracy or predictive performance.

Bone marrow aspirates and trephine biopsies were obtained bilaterally from the right and left posterior iliac crests at baseline before study enrolment and at subsequent protocol-specified timepoints. Thus, most patients contributed serial bone marrow assessments during the study. Repeated examinations were retained as separate observations in the method comparison analyses; right- and left-sided results were combined at the examination level, as described in Section 2.5. For each pairwise comparison, only observations with results available for both methods were included.

Negative control samples were obtained from two patients in whom neuroblastoma was excluded. Clinical and diagnostic data, including results of 123I-MIBG scintigraphy and histopathological bone marrow assessment, were retrieved from the hospital electronic medical record system for comparison with flow cytometry findings. 123I-MIBG scintigraphy uses iodine-123 metaiodobenzylguanidine, which is taken up by neuroblastoma cells and detected using a gamma camera.

Histopathological assessment was based on the final routine reports from bilateral trephine biopsies. The biopsies were evaluated using routine histopathological procedures, including hematoxylin–eosin staining and immunohistochemistry, which was performed in all patients as part of standard diagnostic practice. Markers documented in the available reports included CD56 and synaptophysin. Histopathology was classified as positive when the pathology report documented neuroblastoma cells or an infiltrate immunophenotypically compatible with neuroblastoma, including morphologically characteristic neuroblastoma cells with CD56- and/or synaptophysin-positive staining. The extent of neuroblastoma infiltration was not assessed using a predefined uniform quantitative scoring system in the present analysis because the assessment was based on routine pathology reports. Because trephine biopsies were obtained bilaterally, two biopsy specimens were evaluated per examination when representative material was available. Biopsies reported as non-representative were classified as non-evaluable and were excluded from comparisons involving histopathology.

The study was approved by the Bioethics Committee of the Jagiellonian University (1072.6120.176.2021; 29 September 2021) and conducted in accordance with the Declaration of Helsinki. Written informed consent was obtained from the patients’ parents. All procedures were performed in accordance with relevant guidelines and regulations.

### 2.2. Cytomorphology

Bone marrow smears were prepared during the bone marrow aspiration procedure. At each bone marrow examination, eight smears were prepared, including four from each iliac crest. The smears were stained using the May–Grünwald–Giemsa method according to the Pappenheim procedure. Briefly, the slides were immersed in May–Grünwald solution for 5 min, rinsed with water, and subsequently stained for 10 min with Giemsa solution diluted 1:3 in phosphate buffer. After rinsing and air-drying, the smears were examined through light microscopy. The cellular composition of each bone marrow preparation was assessed by counting up to 1000 consecutive nucleated cells using a manual tally counter. When sufficient cellularity was available, morphologically identifiable neuroblastoma cells were quantified as a percentage of the evaluated nucleated cells; otherwise, their presence was recorded qualitatively as negative (0) or positive (1). The presence of isolated tumor cells, tumor cell clusters, and Homer Wright rosettes was additionally recorded. Cytomorphology was classified as positive when morphologically identifiable neuroblastoma cells, occurring individually or arranged in clusters or rosettes, were detected.

### 2.3. Bone Marrow Processing

Bone marrow aspirates collected in BD Vacutainer^®^ EDTA tubes were processed within 2 h of collection and were used to assess cell concentration using a hematology analyzer Sysmex XN-350 (Sysmex Corporation, Kobe, Japan) and for further staining. From each sample, 50 µL of bone marrow was used for direct analysis, and the remaining sample underwent density gradient centrifugation. Four milliliters of Pancoll (PAN-Biotech GmbH, Aidenbach, Germany) were placed into Falcon tubes. Bone marrow samples, diluted two-fold with saline, were layered onto Pancoll and centrifuged for 30 min at 300× *g* at room temperature. Mononuclear cells (lymphocytes and monocytes) were collected from the interface, while granulocytes and erythrocytes were collected as a pellet. Both cell populations were washed with saline and centrifuged at 400× *g* for 5 min. The cells were resuspended in 250 µL of PBS (BD CellWASH, BD Biosciences, San Jose, CA, USA) for subsequent staining.

**Figure 1:**
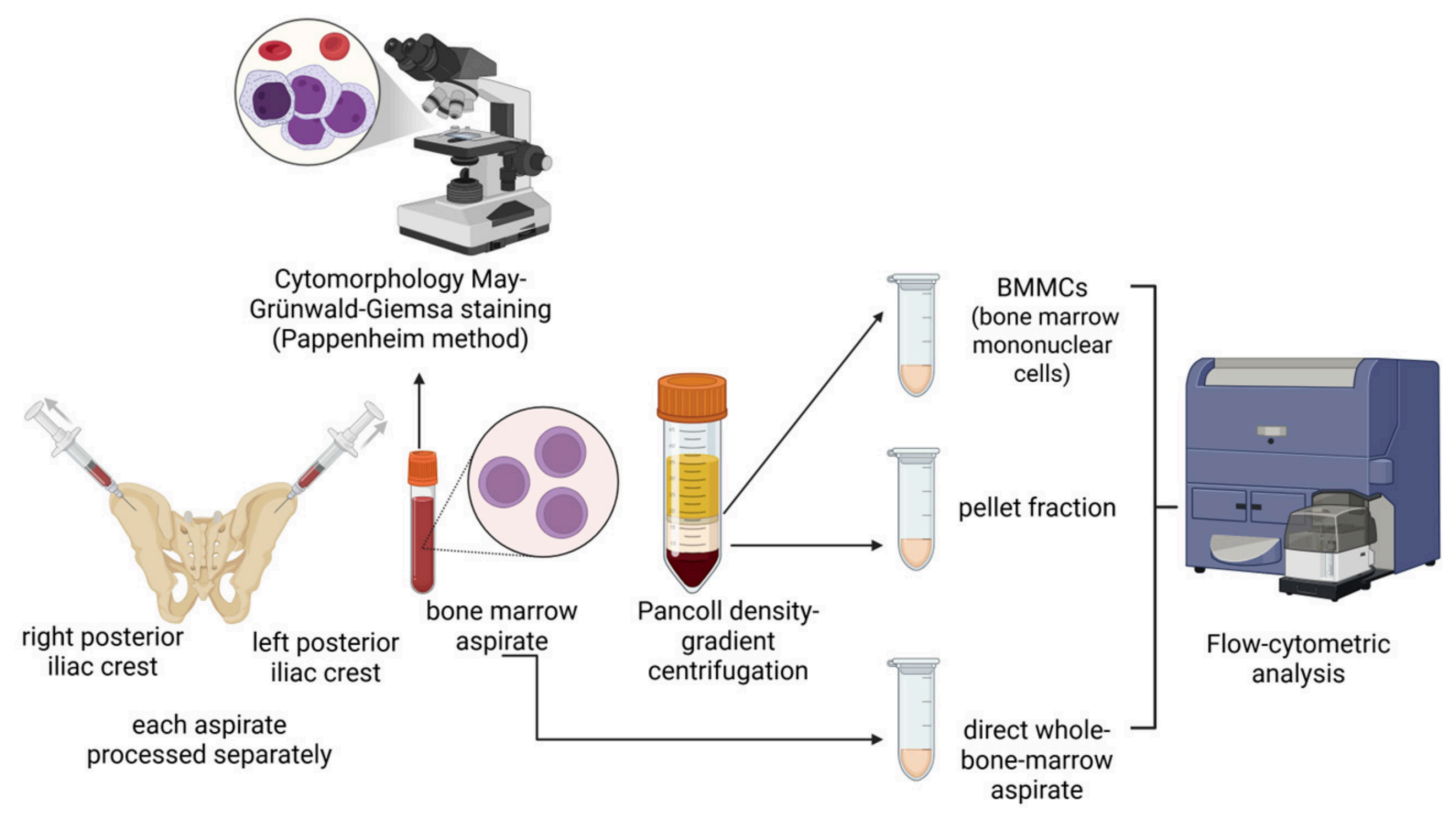
Overview of the study workflow. Bilateral bone marrow aspirates obtained from the right and left posterior iliac crests were processed separately. Cytomorphological assessment was performed on May–Grünwald–Giemsa-stained smears using the Pappenheim method. For flow cytometric analysis, each aspirate was assessed directly as whole bone marrow and after Pancoll density gradient centrifugation as the bone marrow mononuclear cell (BMMC) and pellet fractions. Each fraction was analyzed separately through flow cytometry.

The direct bone marrow aspirate, the mononuclear cell fraction, and the pellet fraction were stained and analyzed separately; fractions were not pooled before flow cytometric assessment. Post-centrifugation cell recovery and viability were not quantified using Trypan blue or a dedicated viability dye. SYTO41 was used to identify nucleated events during flow cytometric gating and was not used as a viability marker. The simplified diagram is shown in Figure 1.

### 2.4. Staining and Flow Cytometric Analysis

Prepared cells from the pellet, mononuclear cell interface, and direct bone marrow aspirates were stained with anti-CD71-FITC and anti-CD36-FITC (combined in a single exclusion channel), anti-CD56-PE, anti-CD99-APC, and anti-CD45-PerCP monoclonal antibodies according to the manufacturer’s instructions (BD Biosciences, San Jose, CA, USA). The panel was selected to identify the expected CD45-negative/CD56-positive neuroblastoma phenotype while excluding erythroid precursors, which may also express CD56, using CD36 and CD71; SYTO41 enabled restriction of the analysis to nucleated cells. CD99 was included as a differential marker because CD99 expression in CD45-negative non-erythroid cells was considered atypical for neuroblastoma. For each sample, 50 µL of bone marrow or cell suspension was mixed with the antibodies, vortexed briefly, and incubated in the dark for 15 min. Fluorescence-minus-one controls were used to support gate placement. Erythrocytes were subsequently lysed using FACS Lysing Solution (BD Biosciences). The cells were washed with BD CellWASH, centrifuged, resuspended in 250 µL of CellWASH and stained with SYTO41 (Invitrogen, Eugene, OR, USA) to identify nucleated cells. Flow cytometric analysis was performed using a BD FACSCanto II cytometer and BD FACSDiva software. After debris exclusion, singlets were selected using FSC-H versus FSC-W, followed by gating on SYTO41^+^ nucleated cells. Neuroblastoma-compatible cells were identified as CD45^−^CD56^+^CD36^−^CD71^−^ events. A reproducibly detected population exceeding the background observed in non-neuroblastoma controls was classified as positive, and its percentage was calculated relative to all gated SYTO41^+^ nucleated cells.

### 2.5. Statistical Analysis

Pairwise comparisons between diagnostic methods (CYT, FCM, MIBG, and HIST) were performed at the level of individual bone marrow examinations, defined as one patient assessment at a given date. Bilateral aspirates were processed and analyzed separately in the laboratory; however, for the method comparison analyses, an examination was classified as positive for a given method if either side was positive, and one examination-level result per date is presented in Table S1. For each pair of methods, overall percentage agreement was calculated as the proportion of concordant positive and negative results. Agreement beyond chance was assessed using Cohen’s kappa coefficient (κ), interpreted according to the Landis and Koch criteria [22]: 0.00–0.20, slight; 0.21–0.40, fair; 0.41–0.60, moderate; 0.61–0.80, substantial; and 0.81–1.00, almost perfect agreement. Two-by-two contingency tables were constructed for each comparison. Correlation analysis was performed between quantitative results obtained through cytomorphology and FCM in aspirate-level records from the right and left iliac crests, analyzed separately, in which at least one measure was positive (Table S2). Spearman’s rank correlation was calculated as a sensitivity analysis, and confidence intervals for Pearson’s r were calculated using Fisher’s z transformation. All statistical analyses and visualizations were performed using Python version 3.11. Aspirates from the right and left iliac crests were never pooled; side-specific results were used for the correlation analysis and for the fraction analysis. Because most patients underwent repeated examinations and the correlation analysis included aspirates from both iliac crests, individual observations were not fully independent; all analyses should therefore be interpreted as exploratory. Generative artificial intelligence tools were used during manuscript preparation, ChatGPT (GPT-5; OpenAI) for language editing, and all outputs were verified by the authors.

## 3. Results

### 3.1. Cytomorphological and Flow Cytometric Detection of Neuroblastoma Cells

**Figure 2:**
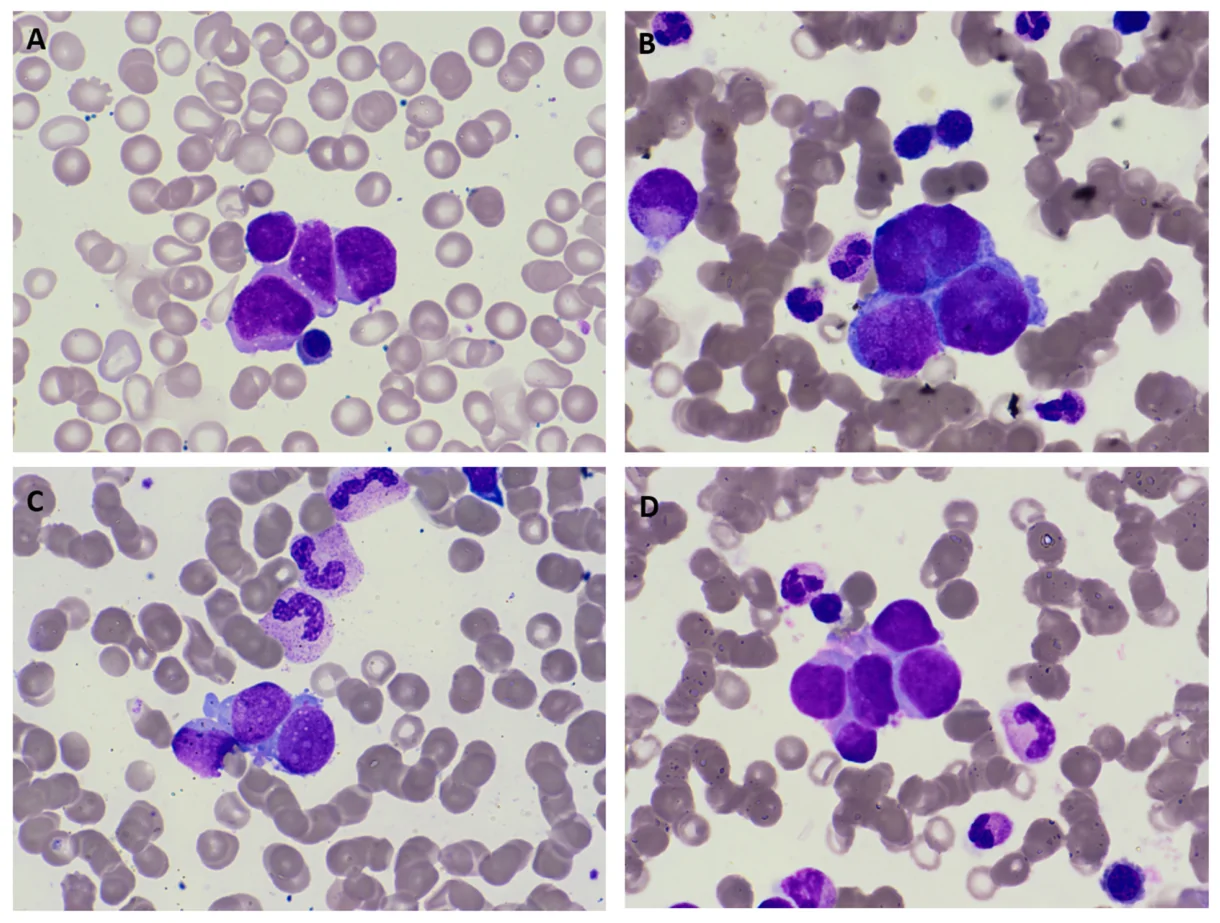
Representative May–Grünwald–Giemsa-stained bone marrow smears from four patients with cytomorphologically positive neuroblastoma involvement. Panels (**A**–**D**) show small clusters of morphologically characteristic neuroblastoma cells among hematopoietic cells. The tumor cells display a high nuclear-to-cytoplasmic ratio, round-to-oval hyperchromatic nuclei, and scant basophilic cytoplasm. Original magnification, 1000× (oil immersion).

Among the 28 patients, bone marrow smears from 6 patients were positive. At the examination level, cytomorphology was positive in 8 out of 91 bone marrow examinations; detailed examination-level results are provided in Table S1. Flow cytometry was positive in 15 out of 90 evaluable examinations from 10 patients. FCM was positive in eight examinations with negative cytomorphology, whereas cytomorphology was positive with negative FCM in one examination. Representative images from four cytomorphologically positive patients are shown in Figure 2.

**Figure 3:**
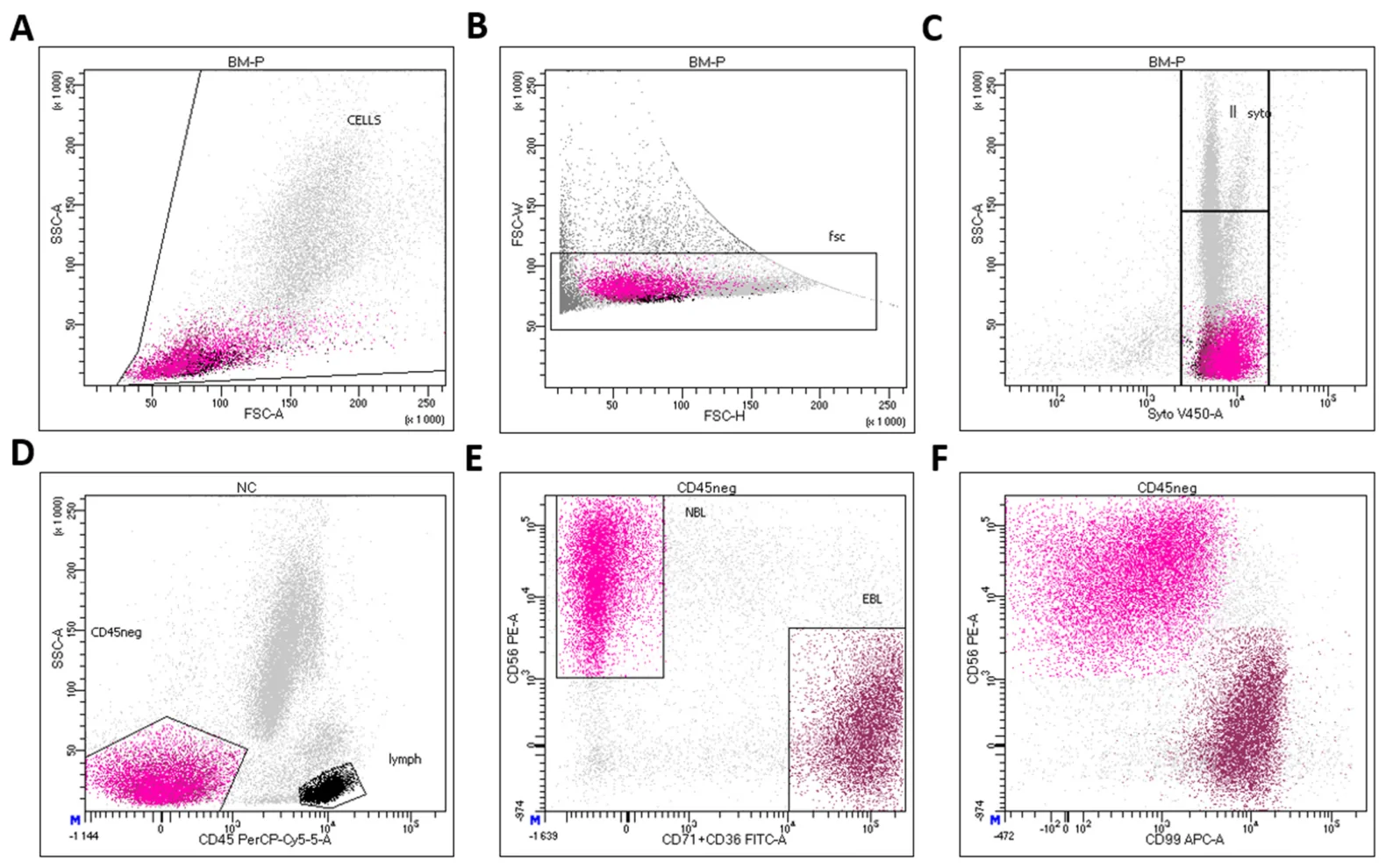
Representative flow cytometric gating strategy for detection of neuroblastoma-compatible cells in a bone marrow aspirate. (**A**) Cellular events were selected after exclusion of debris on the FSC-A versus SSC-A plot. (**B**) Singlets were selected using FSC-H versus FSC-W. (**C**) SYTO41-positive nucleated events were gated. (**D**) CD45-negative events were selected. (**E**) Within the CD45-negative population, erythroid precursors expressing CD36 and/or CD71 were excluded, and neuroblastoma-compatible events were identified as CD56-positive CD36/CD71-negative cells. (**F**) CD99 expression was assessed as a differential marker within the CD45-negative population; CD36/CD71-positive erythroid precursors are shown in dark purple for comparison. Neuroblastoma-compatible cells were expected to be CD99-negative; CD99-positive events were considered phenotypically atypical for neuroblastoma.

The cytometric evaluation was performed on direct whole bone marrow aspirates, the bone marrow mononuclear cell (BMMC) fraction, and the pellet fraction. SYTO41 was used to select nucleated events. Neuroblastoma cells were identified as CD56^+^CD45^−^CD36^−^CD71^−^ events after exclusion of erythroid lineage cells using CD36 and CD71. The representative gating strategy is shown in Figure 3.

### 3.2. Agreement Between Diagnostic Methods

**Figure 4:**
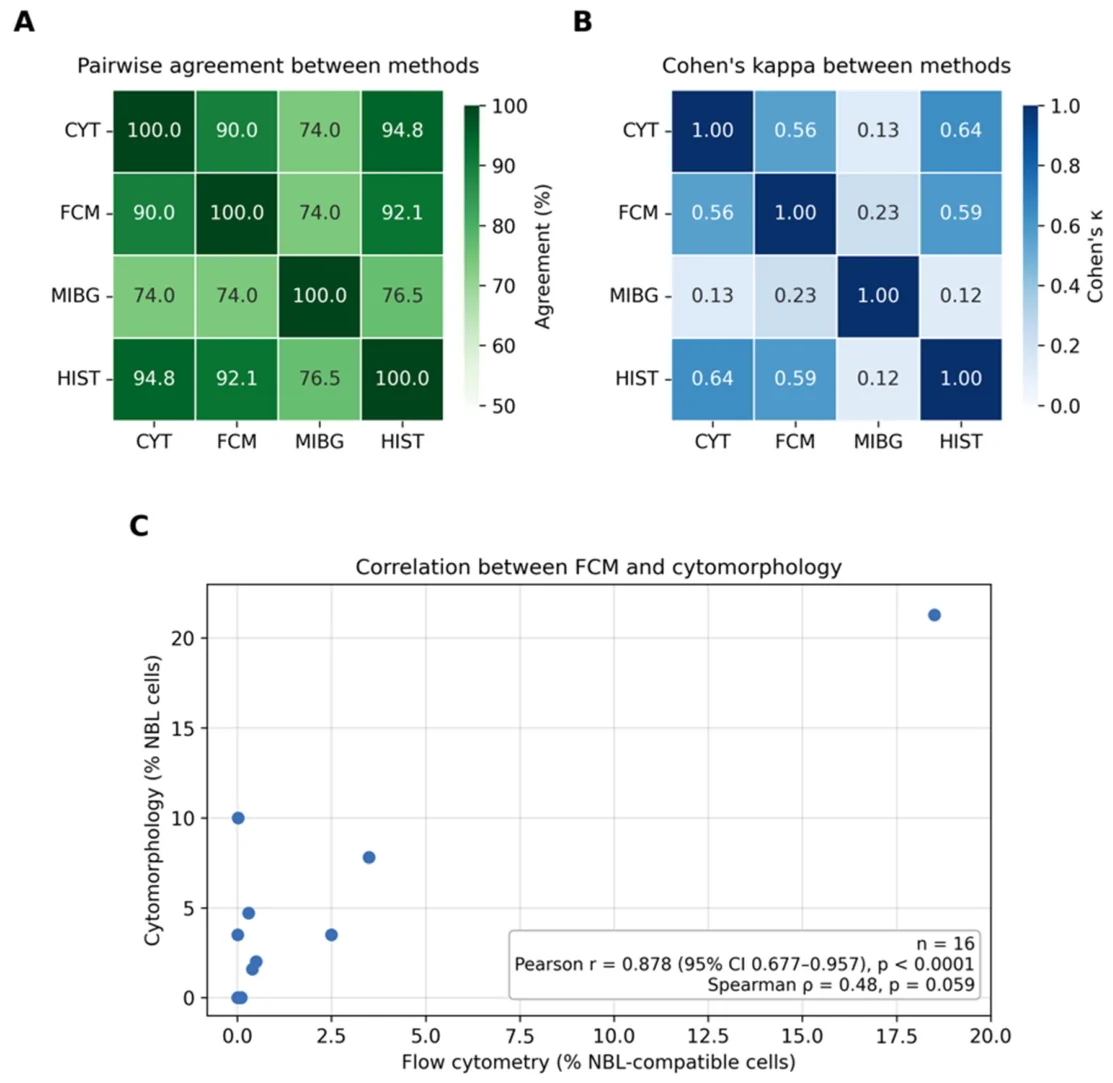
Comparison of results obtained using different diagnostic methods. (**A**) Pairwise agreement (%) between cytomorphology (CYT), flow cytometry (FCM), MIBG scintigraphy (MIBG), and histopathology (HIST). (**B**) Cohen’s kappa coefficients for pairwise comparisons of the same methods. (**C**) Pearson correlation between the whole bone marrow FCM percentage and the percentage of morphologically identifiable isolated NBL cells on cytomorphology in 16 paired aspirate-level records in which at least one measure was positive. Pearson’s r = 0.878 (95% CI, 0.677–0.957; *p* < 0.0001).

Pairwise comparisons between the four diagnostic methods—cytomorphology (CYT), flow cytometry (FCM), 123I-MIBG scintigraphy (MIBG), and histopathology (HIST)—showed 90.0% agreement between CYT and FCM. Agreement between the cell-based methods and HIST was 94.8% for CYT versus HIST and 92.1% for FCM versus HIST. Agreement involving MIBG ranged from 74.0% to 76.5% (Figure 4A; Table 1).

Cohen’s kappa coefficients indicated moderate agreement between CYT and FCM (κ = 0.56) and between FCM and HIST (κ = 0.59) and substantial agreement between CYT and HIST (κ = 0.64). Comparisons involving MIBG showed slight to fair agreement (κ = 0.12–0.23) (Figure 4B). The difference between raw agreement and κ reflects the low proportion of positive examinations, which increases the agreement expected by chance. Of the 15 FCM-positive examinations, histopathology was positive in 5, negative in 6, non-evaluable in 1, and unavailable in 3.

Among 16 paired aspirate-level records in which at least one measure was positive, the whole bone marrow FCM percentage and the cytomorphological percentage of morphologically identifiable isolated NBL cells showed a positive Pearson correlation (r = 0.878, 95% CI 0.677–0.957; *p* < 0.0001) (Figure 4C). This association was sensitive to one high-value observation; Spearman’s ρ was 0.48 (*p* = 0.059), and Pearson’s r was 0.48 (*p* = 0.069) after exclusion of that observation.

### 3.3. Distribution of Neuroblastoma Cells After Density Gradient Separation

Paired numerical results for whole bone marrow and the BMMC fraction were available for 29 samples. The percentage of NBL cells was higher in the BMMC fraction in 6 samples, higher in whole bone marrow in 6 samples, and equal in 17 samples. All samples with large or very large rosettes (categories C and D) had higher NBL cell percentages in the BMMC fraction, whereas all samples with small rosettes (category B) had higher percentages in whole bone marrow. The apparent predominance of equal results in category A was driven partly by samples with 0% NBL cells in both fractions. NBL cells were detected in the bottom fraction in 9 out of 29 evaluable samples, but this fraction never contained the highest percentage. These findings are descriptive because the rosette categories contained few samples (Table 2). Detailed patient-level data and individual sample findings are available in Table S2.

## 4. Discussion

The evaluation of bone marrow involvement in patients with neuroblastoma (NBL) is important for risk stratification and treatment monitoring. Cytomorphological analysis of bone marrow smears and histopathological examination of trephine biopsies remain established components of marrow assessment [14]. Their performance may nevertheless be affected by sample adequacy, focal disease, and the difficulty of detecting low-level involvement. Flow cytometry of bone marrow aspirates may help to address these limitations.

Flow cytometry enables the detection of cells on the basis of predefined antigen expression. In the present cohort, a population of CD45^−^CD56^+^CD36^−^CD71^−^ nucleated events was used to classify a bone marrow aspirate as FCM-positive for neuroblastoma cells. This approach may be particularly useful when the tumor cell frequency is too low for reliable quantitative assessment by cytomorphology [16,23,24,25]. An FCM-positive result indicates the presence of neuroblastoma cells in the analyzed aspirate.

In the International Neuroblastoma Response Criteria, bone marrow involvement of >0% to ≤5% at reassessment is categorized as minimal disease [7,26]. Previous studies have reported variable prognostic relevance of low-level marrow disease detected using sensitive methods [6,20,27,28]. Whether very low-level FCM positivity provides independent prognostic or predictive information will require correlation with treatment outcomes in the planned clinical outcome analysis.

Flow cytometry provides an objective quantitative readout based on predefined marker expression and may reduce some operator-dependent variability [16]. Cytomorphology nevertheless remains essential because it provides direct morphological context, including the recognition of isolated tumor cells, clusters, and rosettes, and enables evaluation of cellular features that cannot be resolved by immunophenotyping alone [29,30]. The two approaches should therefore be regarded as complementary rather than interchangeable. The FCM-derived percentage represents the proportion of recovered CD45^−^CD56^+^CD36^−^CD71^−^ events within the analyzed aspirate and should not be equated directly with the extent of infiltration in a trephine biopsy. Focal and uneven marrow involvement, dilution of the aspirate with peripheral blood, loss of tumor cell clusters during processing, and differential recovery after density gradient separation may all influence the FCM result. FCM findings should therefore be reported as a sample-specific cell fraction and interpreted together with bilateral cytomorphology and trephine biopsy findings rather than converted into a percentage of total marrow infiltration. Future multicenter studies should evaluate how quantitative FCM results relate to standardized bilateral cytomorphological and histopathological assessments using the EuroFlow Solid Tumor Orientation Tube as a basis for harmonized pre-analytical and analytical procedures while validating neuroblastoma-specific quantitative thresholds for serial bone marrow assessment [31].

Although the present targeted panel was not designed as the EuroFlow Solid Tumor Orientation Tube (STOT), it shares core orientation markers, including CD45, CD56, and CD99. In contrast to the broader STOT approach, CD36 and CD71 were included specifically to exclude erythroid precursors, which may otherwise complicate identification of CD45-negative/CD56-positive events in bone marrow aspirates [31].

Comparison of flow cytometry with histopathology showed discordant findings. Of the 15 FCM-positive examinations, 5 had a corresponding positive histopathological result, 6 had a negative histopathological report, the trephine biopsy was non-evaluable in 1, and histopathological data were unavailable for 3. In addition, histopathological assessment was non-representative in 5 out of 91 examinations (5.5%). These findings show that FCM may detect neuroblastoma cells in aspirates when the corresponding trephine biopsy is negative or non-representative, consistent with previous reports [20,32]. Because the two methods assess different specimen types, these discordances do not permit formal estimation of their relative sensitivity or specificity.

Comparisons with 123I-MIBG scintigraphy should be interpreted descriptively only. Of the 91 examinations, 18 had no corresponding MIBG result available for comparison. Of the 15 FCM-positive examinations, MIBG data were available for 12; pathological MIBG uptake was reported in 6 and no pathological uptake in 6. Reported MIBG abnormalities included skeletal lesions in the pelvis and at extra-pelvic sites, including the femur and skull, as well as soft tissue lesions (Table S2). Conversely, FCM-negative iliac crest aspirates occurred in the presence of both pelvic skeletal lesions and lesions at distant skeletal sites. These findings illustrate the focal nature of disease and should not be interpreted as direct discordance between methods. Planar whole-body 123I-MIBG scintigraphy provides the basis for Curie or SIOPEN scoring [12,33], while SPECT/CT may improve the anatomical localization of equivocal MIBG-avid foci. PET/CT with catecholaminergic tracers, such as 18F-DOPA, may improve lesion detection in selected settings [34], whereas 18F-FDG PET/CT is recommended for MIBG-non-avid disease [26]. Whole-body imaging maps the anatomical distribution of lesions, whereas FCM quantifies tumor cells recovered from an aspirate obtained from a single posterior iliac crest sampling site. Consequently, a negative aspirate does not exclude marrow disease at other skeletal sites, and a positive scan does not necessarily establish marrow infiltration at the aspiration site. Because the interval between imaging and marrow examination and lesion location relative to the aspiration site were not standardized, these comparisons cannot estimate the relative sensitivity of the two methods.

The study also explored the distribution of NBL cells within bone marrow fractions obtained through density gradient separation. While no definitive relationship between the form of NBL cells (single cells or clusters) and their distribution among fractions was established, the findings highlighted the technical challenges of flow cytometry in analyzing large cell clusters. Immunocytochemistry, which preserves morphological context, may therefore remain useful in this setting.

This study has several limitations. The cohort was relatively small, and missing comparison data limited direct pairwise analyses between methods. Most patients underwent repeated examinations; therefore, the examination-level analyses did not fully account for within-patient dependence. Although evaluation of bone marrow assessment methods and their relationship with treatment outcome constituted study endpoints, the present report was a methodological exploratory analysis and was not powered to provide definitive estimates of diagnostic sensitivity, specificity, or predictive performance. An independent reference standard was not available for adjudication of discordant findings between methods. MIBG findings were analyzed as whole-body results, and a systematic site-specific analysis relative to the aspiration site was not possible with the available data. The study was not designed or powered to assess associations between FCM findings and clinical stage, INRG risk group, MYCN status, or treatment phase; these associations will be assessed after completion of the planned outcome analysis. The control group was also limited. Finally, external validation in larger cohorts is required. Harmonized pre-analytical and analytical procedures, including those developed within the EuroFlow framework, may facilitate inter-laboratory reproducibility; however, neuroblastoma-specific thresholds for quantitative bone marrow FCM require separate validation.

## 5. Conclusions

Flow cytometry enabled rapid, objective, and quantitative detection of neuroblastoma cell populations in bone marrow aspirates and showed high raw agreement, but only moderate agreement beyond chance, with cytomorphology. It may be particularly useful when low-level involvement is suspected or when conventional specimens are non-representative. The current data do not support replacing cytomorphology, histopathology, or 123I-MIBG scintigraphy because these methods assess different features and anatomical compartments. Larger, preferably multicenter, prospective studies using standardized panels, predefined positivity thresholds, and analyses accounting for repeated measurements are needed to define the clinical performance and role of FCM in neuroblastoma staging and disease monitoring.

## Figures and Tables

**Table 1:** Pairwise agreement and Cohen’s kappa coefficients between diagnostic methods for bone marrow involvement in neuroblastoma: examination-level analysis.

Cohen’s κ	Overall Agreement (%) (n/N)	Method Comparison
0.56	90.0% (81/90)	CYT vs. FCM
0.13	74.0% (54/73)	CYT vs. MIBG
0.64	94.8% (73/77)	CYT vs. HIST
0.23	74.0% (54/73)	FCM vs. MIBG
0.59	92.1% (70/76)	FCM vs. HIST
0.12	76.5% (52/68)	MIBG vs. HIST

Abbreviations: CYT, cytomorphology; FCM, flow cytometry; MIBG, 123I-MIBG scintigraphy; HIST, histopathology. N denotes examinations with results available for both methods, and n denotes concordant examinations. Non-representative trephine biopsies were excluded from comparisons involving HIST. Repeated examinations from individual patients were included; therefore, observations were not fully independent.

**Table 2:** Distribution of NBL cell recovery between whole bone marrow and the BMMC fraction according to rosette size category.

Rosette Category	Evaluable Samples, n	Any NBL-Positive Fraction, n	BMMC > BM, n	BM > BMMC, n	Equal, n	Bottom Positive, n
A, no rosettes	21	8	2	2	17	3
B, small	4	4	0	4	0	2
C, large	3	3	3	0	0	3
D, very large	1	1	1	0	0	1
Total	29	16	6	6	17	9

BM, whole bone marrow; BMMC, bone marrow mononuclear cell fraction. Three samples without paired numerical BM and BMMC values were excluded. The bottom fraction was positive when its NBL cell percentage was greater than zero; it was never the fraction with the highest percentage.

## Data Availability

The data presented in this study are available in the Supplementary Materials and from the corresponding author upon reasonable request.

## Supplementary Materials

The following supporting information can be downloaded at https://www.mdpi.com/article/10.3390/cancers18203355/s1, Table S1: Summary of cytomorphological, cytometric, MIBG, and histopathological evaluation results; Table S2: Side-specific quantitative results for selected examinations.
