# Supplementary material for: Flow Cytometric Detection of Bone Marrow Cell Populations Phenotypically Compatible with Neuroblastoma: An Exploratory Comparison with Conventional Methods
Source: Cancers (Basel). 2026 Oct 16;18(20):3355. doi: 10.3390/cancers18203355 (PMC13702433; doi:10.3390/cancers18203355)
Supplement: Supplementary file 1 [file cancers-18-03355-s001.zip › cancers-4599389-supplementary.pdf]

Table S1. Summary of Cytomorphological (CYT), Cytometric (FCM), MIBG, and Histopathological Evaluation Results (HIST). Legend green: negative result (no lesions/dissemination), red: Positive result (presence of lesions/dissemination), yellow: no data available, * non-representative biopsy.

Patient No.	Date	CYT	FCM	MIBG	HIST
1	07.07.2022	N	N	N	N
1	19.09.2022	N	N	N	N
1	24.05.2023	N	N	N	N
1	17.01.2024	N	N	N	N
2	15.07.2022	N	N	ND	ND
2	14.09.2022	N	N	Partial regression of changes in skull bones	N
2	11.01.2023	N	N	N	N
2	13.03.2023	N	N	N	N
2	23.05.2023	N	N	N	N
2	28.12.2023	N	N	N	N
3	27.12.2022	N	N	N	N
3	24.02.2023	N	N	N	N
3	19.04.2023	N	N	N	N
3	27.06.2023	N	N	N	N
3	08.04.2024	N	N	N	N
4	29.04.2022	P	P	ND	ND
5	21.07.2022	N	N	N	ND
5	13.09.2022	N	N	ND	N
5	14.12.2022	N	N	N	N
5	03.07.2023	N	N	N	N
5	09.01.2024	N	N	N	N
6	24.03.2022	N	P	N	P*
6	30.03.2022	N	N	ND	ND
6	13.06.2022	N	P	N	N
6	31.08.2022	N	P	N	N
6	01.03.2023	N	N	N	N
6	30.06.2023	N	N	N	N
7	19.12.2022	N	N	ND	N

Patient No.	Date	CYT	FCM	MIBG	HIST
7	08.02.2023	N	ND	ND	N
7	12.10.2023	N	N	N	N
7	04.06.2024	N	N	N	N
8	13.05.2022	P	P	P Full bone dissemination	P
9	20.01.2023	P	P	P	N
10	14.09.2022	N	N	ND	N*
10	25.05.2022	N	N	ND	N
10	15.03.2023	N	N	ND	N
10	21.09.2023	N	N	ND	N
11	16.11.2022	N	N	P; lesion present	N*
11	29.07.2022	N	P	P; lesion present	ND
11	20.09.2022	N	N	ND	N*
12	20.07.2022	P	P	ND	P
12	29.09.2022	N	N	ND	N
12	17.01.2023	N	N	N	N
12	22.06.2023	N	N	N	N
12	18.01.2024	N	N	N	N
13	10.05.2022	N	N	P; lesions present	N
13	25.05.2022	N	N	N	N
13	01.08.2022	N	N	P; lesions present	N
14	16.03.2023	N	N	N	N
14	30.05.2023	N	N	N	N
14	28.09.2023	N	N	N	N
14	09.04.2024	N	N	N	N
14	08.07.2024	N	N	N	N
15	15.04.2022	N	N	P; lesions present	N
16	07.02.2023	N	N	ND	ND
16	25.04.2023	N	N	ND	ND
17	03.04.2023	P	P	P	P
17	12.06.2023	P	P	N	N

Patient No.	Date	CYT	FCM	MIBG	HIST
17	31.08.2023	P	N	N	N
17	24.06.2024	N	P	P	N
17	30.09.2024	N	N	P	ND
18	24.05.2023	N	P	P	N
18	17.01.2024	N	N	N	N
19	09.03.2023	N	N	N	N
19	22.05.2023	N	N	N	N
20	07.07.2023	N	N	P	N
20	02.02.2024	N	N	ND	N*
20	08.04.2024	N	N	ND	N
20	08.07.2024	N	N	N	N
20	29.10.2024	N	N	N	N
21	15.01.2024	N	N	N	N
21	18.03.2024	N	N	N	N
21	22.04.2024	N	N	N	N
21	17.12.2024	N	N	N	N
21	12.03.2025	N	N	N	N
22	09.08.2024	N	N	P	N
22	14.06.2025	N	N	N	N
22	24.03.2025	N	N	N	N
23	13.06.2024	P	P	N	P
23	04.09.2024	N	P	N	P
24	18.11.2024	N	N	N	N
24	16.01.2025	N	N	N	N
25	26.02.2025	N	N	P	N
25	24.12.2024	N	N	P	N
25	18.02.2025	N	N	P	N
26	03.01.2025	N	N	N	N
26	17.03.2025	N	N	P	N
26	25.07.2025	N	N	P	N
27	22.03.2023	N	N	N	N

Patient No.	Date	CYT	FCM	MIBG	HIST
27	06.06.2023	N	N	N	N
28	26.04.2023	N	P	ND	ND

Table S2. Side-specific quantitative results for selected examinations. RS, right iliac crest; LS, left iliac crest; BM%, percentage of NBL cells in whole bone marrow; BMMCs%, percentage of NBL cells in the bone marrow mononuclear-cell fraction; Base%, percentage of NBL cells in the pellet fraction; % of single cells, percentage of NBL cells not occurring in clusters on cytomorphological examination; – or empty cell, no result owing to insufficient material. Rosette density indicates the number of rosettes in the smear, graded 0–4: 0, no rosettes; 1, few; 2, numerous; 3, abundant; 4, very abundant. Rosette size indicates the average number of cells per rosette, graded A–D: A, no rosettes; B, small rosettes (3–6 cells); C, large rosettes (up to approximately 20 cells); D, very large rosettes (up to several dozen cells).

Patient No.	Examination Date	BM%	BMMCs %	Base%	% of Single Cells	Rosette Density	Rosette size	MIBG	Trepanobiopsy
4 RS	29.04.2022	18.5	26.8	1.5	21.3	4	D	No data	No data
4 LS		-	-	-	4.3	3	C		No data
6 RS	24.03.2022	0.1	0.1	0	0	0	A	No lesions	Very sparse population of neuroblastoma cells present in the marrow, a single large CD56+ cell identified
6 LS		0.1	0.1	0	0	0	A		Very sparse population of neuroblastoma cells present in the marrow, a few elongated, scattered CD56+/synaptophysin+ cells
8 RS	13.05.2022	0.5	0	0	2	1	B	Full skeletal dissemination	Presence of a small neuroblastoma cell infiltrate, <5% CD56+ cells
8 LS		0.4	0	0	1.6	1	B		Negative
9 RS	20.01.2023	0.3	0.4	0.2	4.7	2	C	A subpleural, right parasternal tissue lesion with mildly increased tracer uptake - suspected proliferative nature	negative
9LS		0	0	0	0	0	A		negative
11 RS	29.07.2022	0.01	0	0	0	0	A	Pathological changes persist in C7 vertebra,	No data

								hip bone, and cranial bone	
11 LS		0.07	0.1	0.01	0	0	A		No data
12 RS	20.07.2022	3.5	5.9	0.9	7.8	2	C	No data	Atypical CD56+ cells constitute 50% of marrow cells in the biopsy
12 LS		2.5	5.1	0.5	3.5	2	C	No data	As above
13 RS	10.05.2022	0	0	0	0	0	A	Metastatic changes in femoral and cranial bones	Negative
13 LS		0	0	0	0	0	A		Negative
15 RS	15.04.2022	0	0	0	0	0	A	Pathological tracer accumulation in the right ischial bone	Negative
15 LS		0	0	0	0	0	A		Negative
17RS	03.04.2023	0	0	0	0	0	A	Bone lesions with trace tracer uptake	negative
17LS		0.02	0.01	0.003	10	2	B		Bone marrow involvement by 10% CD56+ atypical cells with hyperchromatic nuclei and pale cytoplasm in irregular scattered nests
18RS	24.05.2023	0.011	0.016	0.003	0	0	A	uptake in the tumor located in the left thoracic inlet	negative
18LS		0.062	0.024	0.043	0	0	A		negative
20RS	07.07.2023	0	0	0	0	0	A	Right adrenal tumor	negative
20LS		0	0	0	0	0	A		negative
22RS	09.08.2024	0	0	0	0	0	A	Solitary occipital lesion	negative
22LS		0	0	0	0	0	A		negative
23RS	13.06.2024		0	0	0	0	A	Negative	Presence of large atypical CD56+ cells with moderately abundant fibrillar cytoplasm, forming focal sheet-like aggregates
23LS		0.01	0.008	0.001	3.5	2	B	negative	Presence of CD56+ atypical cells, dispersed and arranged in small focal sheet-like aggregates.
25RS	26.02.2025		0	0	0	0	A	Persistent mild pathological tracer uptake in the tumor bed (para-aortic region) and bilaterally in the lower thorax near the diaphragmatic crura. New metastatic	negative

								lesion in the right ischium, an indeterminate focus of tracer uptake in the hepatic hilum	
25LS		0	0	0	0	0	A		negative
26RS	17.03.2025	0	0	0	0	0	A	Uptake in the tumor lesion of the right adrenal gland	negative
26LS		0	0	0	0	0	A		negative
28 RS	26.04.2023	0.1	0.1	0	0	0	A	No result	No result
28LS		0.1	0.1	0	0	0	A		No result
